# Beyond the Gut: A Systematic Review of Oral Manifestations in Celiac Disease

**DOI:** 10.3390/jcm12123874

**Published:** 2023-06-06

**Authors:** Alberta Lucchese, Dario Di Stasio, Simona De Stefano, Michele Nardone, Francesco Carinci

**Affiliations:** 1 Multidisciplinary Department of Medical-Surgical and Dental Specialties, University of Campania “Luigi Vanvitelli”, 80138 Naples, Italy; 2 Ministry of Public Health, 00144 Rome, Italy; 3 Asst Melegnano Martesana, Regione Lombardia, Adda District, 20077 Vizzolo Predabissi, Italy; 4 Department of Translational Medicine, University of Ferrara, 44121 Ferrara, Italy

**Keywords:** celiac disease, gluten, oral manifestations

## Abstract

Background: Celiac disease (CD) is a chronic immune-mediated gluten-sensitive enteropathy, affecting about 1% of the population. The most common symptoms include diarrhea, abdominal pain, weight loss, and malabsorption. Extra-intestinal symptoms include oral manifestations. This systematic review aims to catalog and characterize oral manifestations in patients with CD. Methods: a systematic literature review among different search engines using PICOS criteria has been performed. The studies included used the following criteria: tissues and anatomical structures of the oral cavity in humans, published in English and available in full text. Review articles and papers published before 1990 were excluded. Results: 209 articles were identified in the initial search. In the end, 33 articles met the selection criteria. The information extracted from the articles was classified based on the type of oral manifestation. Recurrent aphthous stomatitis (34.6%), atrophic glossitis and geographic tongue (15.26%), enamel defects (42.47%), delayed dental eruption (47.34%), xerostomia (38.05%), glossodynia (14.38%), and other manifestations including cheilitis, fissured tongue, periodontal diseases, and oral lichen planus were found in the celiac subjects of the studies analyzed. The quality of articles on the topic should be improved; however, oral manifestations in CD patients are widely described in the literature and could help diagnose celiac disease.

## 1. Introduction

Celiac disease (CD) is an enduring immune-mediated enteropathy that is characterized by an immunological response to gluten, a protein complex present in wheat, barley, and rye [1]. This condition is attributed to the proteins, namely alpha gliadins, and glutenins in wheat, which may incite a toxic event in the intestinal mucosa of genetically predisposed individuals. The result is often villous atrophy and lymphocyte infiltration in the small intestinal mucosa [2]. Although the exact global prevalence of CD remains uncertain, it is estimated to affect approximately 1% of the population worldwide [1]. Notably, non-celiac gluten sensitivity (NCGS) is also recognized and is defined by symptoms akin to celiac disease. These symptoms improve after gluten is excluded from the diet and in the absence of serological or histological evidence of celiac disease [3].

CD diagnosis frequently occurs in individuals with a familial history of the condition or other autoimmune disorders. Typically, the diagnostic process involves a combination of serological testing, genetic testing, and small intestine biopsy [4]. Genetically, most patients with CD express HLA-DQ2 or HLA-DQ8 haplotypes, although these are also found in approximately 30–40% of the general population [5,6].

Clinical manifestations of CD are wide-ranging. Some patients may be asymptomatic, while others may experience severe symptoms [7]. The most frequently reported symptoms are diarrhea, abdominal pain, weight loss, and malabsorption [8]. However, this disease can also present through many extraintestinal manifestations, including those in the oral cavity. While CD primarily affects the proximal part of the intestinal mucosa, evidence suggests that gluten-driven T cell activation propagates throughout the entire gastrointestinal (GI) tract [9]. Given that the mouth is the initial segment of the GI system, it can provide a readily detectable site where manifestations of CD can contribute significantly to diagnostic processes [10]. Both hard and soft oral tissue lesions have been associated with CD, and the co-occurrence of aphthae with CD could indicate underlying autoimmune reactions [11]. Common oral manifestations of CD encompass recurrent aphthous ulcers (RAS), dental enamel defects (DED), delayed dental eruption, multiple caries, angular cheilitis, atrophic glossitis, dry mouth, and a burning sensation in the tongue [12,13].

When individuals with CD ingest gluten, their immune response leads to inflammation and damage within the small intestine. This damage, known as villous atrophy, precipitates nutrient malabsorption, which can manifest as diverse symptoms, including RAS and DEDs [14]. Notably, DEDs are observed with higher frequency in individuals with CD as compared to the general population.

The precise mechanisms linking CD to DEDs remain elusive. Ongoing research is exploring the potential roles of nutritional, genetic, and immunological factors in interrupting the normal course of amelogenesis. Certain factors, such as calcium, phosphate, and vitamin D malabsorption, might impact amelogenesis. Comparative studies have identified discrepancies in serum calcium levels between patients with CD and non-CD individuals [15,16].

The exact mechanism associating RAS with CD is yet to be fully elucidated. However, it is speculated to be connected to the immune response elicited by gluten. Investigations have revealed elevated inflammatory markers and antibodies in individuals with CD and gluten-sensitive enteropathy (GSE), which might contribute to the onset of RAS [17] (Supplementary Materials).

It is worth noting that RAS is not a specific or characteristic symptom of CD. These ulcers can be associated with other medical conditions, such as inflammatory bowel disease and Behçet’s disease [18].

This systematic review aims to identify and categorize oral manifestations in patients diagnosed with CD.

## 2. Materials and Methods

We performed a systematic review of the literature using different search engines (PubMed, ISI Web of Science, and Cochrane Library). The employed MeSH terms were as follows: celiac disease, gluten-sensitive enteropathy, oral manifestation, oral lesion, oral complication, oral disorder, oral mucosa, and oral mucosal disease. Search operations ended in February 2023. The review was performed following the PICOS criteria and PRISMA checklist. The populations of interest were male and female patients, of all ages, with confirmed diagnoses of celiac disease, characterized by alterations of the soft and hard tissues of the oral cavity (intervention); the comparison was no intervention. Study designs included case-control studies, cross-sectional studies, retrospective and prospective cohort studies, survey studies, case series, and case reports. Our aim was to identify and classify oral manifestations in CD. The included criteria of the examined articles were presence of manifestations in tissues and anatomical structures of the oral cavity in humans, published in English and available full text. Review and articles published before 1990 were excluded. The selection took different steps: after collecting all the initial results, three reviewers (DDS, AL, and FC) read the titles and abstracts, excluded duplicates, and ruled out all those articles that did not meet the inclusion criteria during this initial analysis. Then, two reviewers (DDS and AL) read the full texts of the remaining articles in depth to better evaluate the content. Quality assessment of non-randomized studies will be based on the Risk of Bias in Non-randomized Studies of Interventions (ROBINS I) assessment tool [19]. This tool evaluates seven bias domains, and each one refers to the risk of bias (RoB) in five grades: low, moderate, serious, critical, and no information. The overall evaluation is based on the combination of these seven domains. A study based on a non-randomized design rarely presents a low level of RoB.

The review was submitted and registered on PROSPERO (CRD42023390902).

## 3. Results

**Figure 1:**
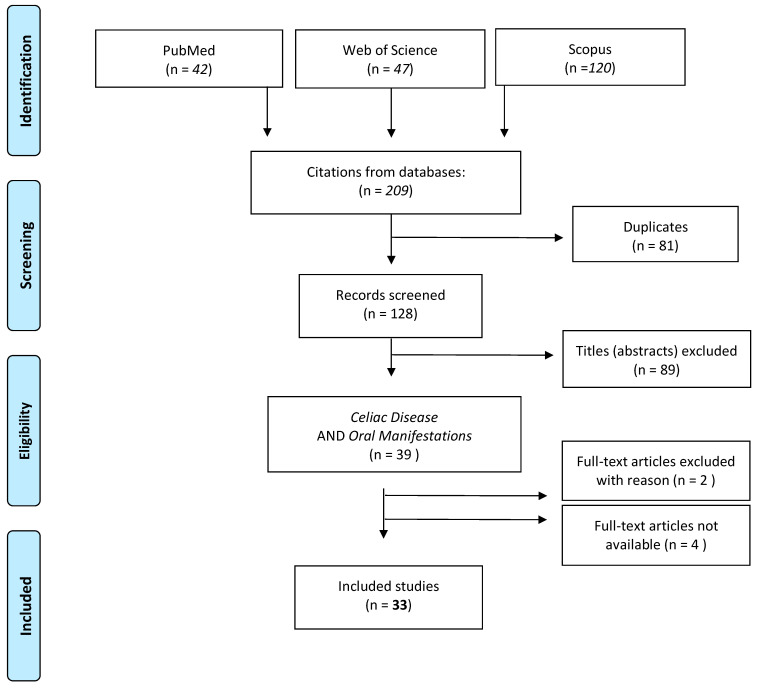
The PRISMA flow diagram summarizing the steps in the selection process.

A total of 209 articles were identified in the initial search. Of those, 81 were duplicates, and 128 were original articles. Among these, 89 did not match our selection criteria. Reading the full-text version led to the exclusion of further six articles. In the end, we identified 33 articles that met our criteria (Figure 1). The PICOS information about the 33 articles and their main contents is summarized in Table 1, Table 2, Table 3, Table 4, Table 5, Table 6 and Table 7.

These 33 items, selected by the type of oral manifestation found in our research, were divided into 7 groups.

The total population studied comprised 1913 subjects with CD. For 1750 patients (66.6% of the articles selected), it was possible to calculate the total gender distribution: 66.06% of patients were female, while 33.94% were male.

### 3.1. Risk of Bias in Individual Studies

The evaluation of 33 non-randomized studies using the ROBINS-I assessment tool showed that 8 studies were rated as having a moderate risk of bias, while 25 were rated as having a low risk of bias. The primary reasons for the low risk of bias were selection bias (no studies included volunteers) and the absence of blinding for participants, healthcare providers, and outcome assessors; the moderate risk of bias was assessed on the limited number of patients included in eight of those studies. Despite the moderate presence of bias, the studies still provide credible evidence, and none were found to have a critical level of bias in any domain.

### 3.2. Recurrent Aphthous stomatitis (RAS)

Twenty-seven articles documented patients with RAS or a history of RAS [11,20,21,22,23,24,25,26,27,28,29,30,31,32,33,34,35,36,37,38,39,40,41,42,43,44,45]. These studies encompassed 1352 celiac patients and 1477 healthy individuals. The mean age of the study subjects was 20.35 ± 14.1 years (range 7.5–49.2 years). The overall prevalence of RAS in the study population was 457/1352 (33.8%), while in the control group, it was 190/1477 (12.9%). The data from each of these studies are presented in Table 1.

Two studies [28,32] were case reports featuring adult female patients. Da Silva et al. [28] reported the presence of simultaneous oral ulcers persisting for approximately one month, with extensive pain symptoms across all intraoral mucosal regions. Kovacic et al. [32] detailed a case of a patient with necrotizing ulcerative stomatitis (NUS) and a medical history of RAS. The necrosis advanced rapidly, culminating in a defect that necessitated plastic reconstructive surgery using two flaps from the remaining tissue of the lower lip for compensation.

Of the remaining 25 articles, it is noteworthy that 8 studies [25,27,29,33,35,37,43,44] did not report any significant differences in the presence of RAS between patients with CD and the control group.

Conversely, the other seventeen studies [11,20,21,22,23,24,26,30,31,33,34,36,39,40,41,42,44] presented data indicating that the occurrence of RAS in the study group was statistically significantly higher when compared to the control group.

In the study conducted by Alsadat et al. [22], RAS was detected in 42.3% of children diagnosed with CD and 15.4% of controls (*p* < 0.001). The presence of CD markedly elevated the likelihood of having RAS, showing an approximately fourfold increase compared to healthy controls.

In a study conducted by Yilmaz et al. [45], the frequency of blood deficiencies (anemia, iron, folate, and vitamin B12) and CD in children with RAS was examined. These patients experienced an average of 4 ± 2.4 aphthous ulcers per year (ranging between 3 and 20). Of these, 82% were classified as minor aphthous ulcers, while 18% were identified as herpetiform aphthous ulcers. Only three patients (2.7%) in the RAS group were diagnosed with CD.

Bijelić et al. [23] analyzed the presence and concentrations of specific serological markers for celiac disease (CeD) and inflammatory bowel disease (IBD) in patients with RAS who did not display gastrointestinal symptoms. The authors hypothesized a common etiopathogenesis for RAS, CeD, and IBD: a dysregulation of mucosal immunity. They revealed a higher prevalence and increased IgG concentrations of antibodies against Saccharomyces cerevisiae (ASCA) in RAS patients compared to controls (*p* < 0.01). Moreover, they reported higher concentrations of IgA anti-tTG (*p* = 0.002) and IgA anti-GAF-3X (*p* = 0.04) antibodies in RAS patients compared to healthy volunteers. Nonetheless, only one patient with RAS was diagnosed with CD (Marsh type III).

### 3.3. Atrophic Glossitis and Geographic Tongue

A total of 8 studies highlighted atrophic glossitis or geographic tongue as manifestations of CD [11,24,26,33,35,36,40,41], with a total population of 616 patients with CD and 730 controls. The mean age of the study subjects was 25.69 ± 15.65 years (range 7.5–49.2 years). The overall prevalence of atrophic glossitis and geographic tongue was 15.26% among celiac patients and 4.52% among the controls. The data from these individual studies are detailed in Table 2.

One study by Campisi et al. [26] reported a statistically significant difference for both atrophic glossitis (*p* < 0.0001) and geographic tongue (*p* < 0.0001). However, while the other studies found intraoral soft tissue lesions to be more frequent in the CD group than the control group, the differences were not statistically significant.

### 3.4. Dental Enamel Defects (DEDs)

Nineteen studies reported the occurrence of dental enamel defects (DEDs) [11,20,21,22,24,25,26,27,29,30,31,33,35,37,39,41,42,43,46]. The mean age of the subjects involved in these studies was 16.27 ± 11.01 years (range 7.5–44.6 years). The overall prevalence of DEDs was 42.47% among patients with CD and 15.04% among the controls. The data from these individual studies are presented in Table 3. The severity of enamel defects in CD patients and in controls was evaluated according to Aine’s classification [52].

Only 2 of these 19 papers reported a non-statistically significant association between celiac disease and dental enamel defects. In a cross-sectional prevalence study by Saraceno et al. [42] involving 83 patients with CD, 10 had enamel hypoplasia (12%), versus 5 in the control group (6%). The difference between the two groups was not statistically significant (*p* > 0.05). Elbek-Cubukcu et al. [30] reported that the prevalence of molar–incisor hypomineralization (MIH) was found in 61% of children with CD. The total percentage of children without MIH was 52.0% (n = 65). The rate of children with MIH in the CD group was 37.7% (n = 23), whereas it was 65.6% (n = 42) in the control group. Out of the CD children, 11.7% had MIH > 4, including permanent incisors, while no children with MIH > 4 were in the control group. The study revealed a moderately significant inverse relationship between the number of MIH and the age at diagnosis (r = −0.605, *p* < 0.001) in children with CD. Conversely, a positive association was observed between the duration of CD and the number of MIH (r = 0.536, *p =* 0.001).

### 3.5. Delayed Dental Eruption (DDE)

Four articles reported delayed dental eruption (DDE), including two case-control studies [37,47], one retrospective cohort study [39], and one cross-sectional study [24]. The mean age of subjects was 8.98 ± 1.58 years (range 7.5–10.67 years). The prevalence of DDE was 47.34% in the celiac population and 7.85% in the control group. The data from these individual studies are presented in Table 4.

In the study by Alamoudi et al. [47], children with celiac disease showed a dental development delay (DDM) of 0.66 ± 0.91 years (7.94 ± 10.94 months) when compared to healthy controls, who had advanced dental maturity of 0.58 ± 0.73 years (6.99 ± 8.77 months). This difference was statistically significant (*p* < 0.001).

In contrast, the study by Moreau et al. [39] reported that none of the CD patients (0/28) had DDE, while it was present in 3.57% of cases in the control group. Furthermore, in the sample analyzed by Mina et al. [37], the delayed eruption of temporary and permanent teeth was marginally higher in the control group, but the difference was not statistically significant.

Bramanti et al. [24] found that 19 of the confirmed celiac patients (38%) experienced a delay in dental eruption averaging 1.4 years, while 9 of the potential celiac patients (42.8%) had an average delay of 1.7 years. In contrast, only six healthy individuals in the control group (11.1%) experienced a minor delay in tooth eruption, with an average delay of less than one year.

### 3.6. Xerostomia

Seven studies identified xerostomia as an intraoral manifestation of CD, among which three were case-control studies [27,29,43], three were cross-sectional studies [21,33,34], and one was a structured questionnaire (survey) [40]. The mean age of the subjects was 28.06 ± 18.46 years (range 8.9–49.2 years). The prevalence of xerostomia was 38.05% in the celiac population compared to 8.68% in the control group. The data from these individual studies are shown in Table 5.

Cruz et al. [27] evaluated subjective dry mouth by asking patients to report signs and symptoms and by measuring the flow rate of unstimulated and stimulated saliva. In the study by de Carvalho et al. [29], 18 out of 50 CD patients had a low salivary flow rate (36%) compared to 12% of healthy controls (6/50). CD patients were 9.15 times more likely to report symptoms of dry mouth (*p =* 0.002) than the control group, with 32.5% of the CD group versus 5% of controls reporting these symptoms. According to Liu et al. [34], xerostomia was the most commonly reported oral symptom. Additionally, 20% reported difficulty swallowing dry food and/or speaking due to xerostomia. Signs of dryness were prevalent in the patients’ mucosal tissues, including dry and cracked lips and labial mucosa. However, CD patients showed significantly higher rates of unstimulated and chewing-stimulated whole saliva flow than the healthy control group (*p* = 0.01 and *p* = 0.05, respectively). Although not statistically significant, the flow rate of stimulated parotid saliva was also higher in the patient group (*p* = 0.06).

### 3.7. Glossodynia

Four studies reported glossodynia as an oral manifestation in patients with CD. The mean age was 28.62 ± 20.78 years (range 7.5–72 years). The overall prevalence of glossodynia was 14.38% in celiac patients compared to 5.92% in the control group. The data from these studies are shown in Table 6.

In Bramanti et al.’s study [24], glossodynia was defined as a combination of subjective burning sensations and objective signs of redness and swelling of the papillae at the tip of the tongue unrelated to injury. This symptom was found in 14% of CD patients (7/50), 9.5% of potential CD patients, and 5.55% of controls. The authors suggested that tongue-burning symptoms were mainly associated with the patients’ anemic conditions. Notably, the study population was of pediatric age.

Shahraki et al. [43] detected a burning tongue sensation in 8 out of 65 children with CD (12%), a finding that was not statistically significant compared to the control group (3%, *p* = 0.06).

In the retrospective observational study by Ludovichetti et al. [35], a higher prevalence of glossodynia was found in pediatric patients with CD (6/38—15.8%) compared to patients with gastrointestinal conditions and malabsorption but without a CD diagnosis (4/38—10.5%), and no cases were found in the control group.

Lucchese et al. [48] reported a case of a 72-year-old female subject who had been experiencing a burning sensation on the dorsum of her tongue for four months. The symptoms resolved within one month after the initiation of a gluten-free diet.

### 3.8. Other Oral Manifestations

The reviewed studies reported several other oral manifestations in patients with CD.

Cheilitis, an inflammation of the lips, was reported in five of the studies [21,24,35,36,50], as detailed in Table 7. In the study by Ahmed et al. [21], 13.6% (16/118) of CD patients had cheilosis/angular cheilitis, while none of the controls did. Bramanti et al. [24] noted minor to mild angular cheilitis in 6% of CD patients, 9.5% of potential CD patients, and 3.7% of controls. Ludovichetti et al. [35] reported angular cheilitis in 10.5% of pediatric CD patients and 7.9% of cases with gastrointestinal conditions and malabsorption without a CD diagnosis, with no instances in the control group. In the study by Macho et al. [36], angular cheilitis was more prevalent in the CD group, but this was not statistically significant when compared to controls (*p* = 0.059).

Two studies reported the presence of a fissured tongue (Table 7). In Liu et al.’s study [34], 20% of the study group (4 out of 20 patients) and 2 controls presented with this oral sign (OR = 2.3; 0.4–14.0; *p* = 0.38). In Lucchese et al.’s case report [48], the patient also exhibited tongue fissuring.

Družijanić et al. [49] discovered 7 cases with CD in a population of 63 patients diagnosed with oral lichen planus (OLP). Saalman et al. [51] reported a case of orofacial granulomatosis (OFG) in a CD patient with diabetes mellitus among eight pediatric subjects with OFG and gastrointestinal inflammatory conditions.

In the study by Kustro et al. [50], 20% of patients were diagnosed with inflammatory periodontal diseases and 80% with dystrophic-inflammatory periodontal diseases. These researchers found a high prevalence of dystrophic-inflammatory periodontal diseases in patients with non-celiac gluten sensitivity (NCGS), with 72% of NCGS cases detected. Additionally, Nota et al. [40] reported that 105 out of 237 patients (44.3%) experienced gingival bleeding.

## 4. Discussion

The present systematic work showed an extensive review of all oral manifestations associated with a diagnosis of celiac disease. The present study’s results align with other systematic reviews conducted previously. Nieri et al. [13], a systematic review and meta-analysis conducted on RAS and DED in patients with celiac disease, showed that both manifestations were more frequent in celiac subjects than in healthy controls. However, according to the authors, the subjects’ age may impact the association between CD and DED. The odds ratio for children was similar to the overall ratio, while the adult ratio was lower and not statistically significant. However, since only three studies analyzed celiac adult subjects, the results may not be reliable. A third subgroup that included children and adults had high variability, possibly due to age differences in the studies. These findings suggest that the relationship between celiac disease and enamel defects exists in children but not adults. Furthermore, Souto-Souza et al. [53] also conducted a metanalysis on DED and CD. They found that while patients with celiac disease (CD) were significantly associated with enamel defects in an overall analysis, neither the permanent dentition nor the mixed dentition of CD patients showed an association with enamel defects. Only the group of CD patients with deciduous dentition was found to be associated with DED. However, in three studies [21,26,33] on the adult population, the prevalence of DED in patients with CD turns out to be significantly higher than in controls. A recent paper by Ahmed et al. [21] on 118 CD patients and 40 controls showed that DED was present in 66.9% of adult patients with CD (age range 20–37.23 vs. 21.3–36.8). The correlation between CD and DEDs could involve factors beyond nutritional deficiencies, including the timing of amelogenesis interruption and autoimmune reactions against amelogenins and ameloblastin, which guide enamel mineralization [54]. Sera from CD patients can recognize amelogenins, a prominent component in the enamel matrix; this was further supported by the discovery of high IgG reactivity against gliadin peptides and enamel matrix protein extract in patients with untreated CD, indicating a pathological role for antibodies to gliadin in DEDs.

Moreover, children with untreated CD exhibited higher serum levels of anti-amelogenin IgA, and the ones with the most severe CD displayed higher anti-amelogenin IgG immune reactivity than controls. Detailed IgA anti-amelogenin epitope mapping using selected blood samples from CD children with high IgA anti-amelogenin reactivity revealed that the primary reactivity was directed to specific segments of the amelogenin peptide [55]. These findings suggest the complex interplay of genetic, immunological, and potentially autoimmune factors in developing DEDs in CD patients. The presence or absence of specific human leukocyte antigen (HLA) class II genes can play a role. These include HLA-DQ2.5, -DQ2.2, and -DQ8, and their absence is often utilized to dismiss the possibility of CD. Interestingly, the HLA-DQB1*02 allele has been linked to oral manifestations, such as DEDs [56]. This association has been observed in both adult and pediatric CD patients, suggesting that genetic factors, namely the expression or lack of specific alleles, may play a considerable role in developing DEDs in the context of CD.

Furthermore, concerning RAS, data reported in this review show a higher prevalence in the celiac population than in healthy control; this is in line with Nieri et al. [13], who even report an odds ratio of 47.90 in the adult population compared with the control groups. However, the authors point out that analyzing the prevalence of RAS is challenging because it is affected by the introduction of GFD. Traditionally, it was thought that the GFD was effective in almost all cases of CD, but a substantial portion of patients, especially adults, fail to improve completely [15]. In our study, we reported the results of Macho et al. [36], which not only measured the frequency of occurrence of canker sores in the CD group and the controls but also for 13 patients for whom a reduced frequency of lesions onset from the beginning of GFD administration was prospectively assessed. The same reduction was reported by Yazdamabod et al. [44] in two patients with CD and RAS. Furthermore, Campisi et al. [26] reported that 89% of the patient showed remission of RAS after one year of GFD. Patients with RAS show an imbalance in cytokine production, a condition that could also be noted in CD’s etiopathogenesis. There is an upregulation in the secretion of Th1 cytokines, such as IL-2, IFN-c, and TNF-a, while anti-inflammatory cytokines, such as TGF-b and IL-10, notably decrease. This dysregulation between pro-inflammatory and anti-inflammatory responses may trigger auto-immunization, facilitating the onset of RAS in genetically predisposed individuals [57]. A consistent finding among RAS patients is the increased number of T lymphocytes capable of producing pro-inflammatory cytokines (IL-2, IL-12, and IFN-c), coupled with a decline in the IL-10 producing cells. This skewed balance towards a Th1-type immune response is also witnessed in CD [58].

Notably, lymphocytes expressing T c/d cell receptors, which produce IL-2 and contribute to epithelial growth control, have been found in increased concentrations in individuals with CD [59]. Moreover, a deficiency in hematins, including iron, folic acid, and vitamin B12, has been noted in certain RAS patients [60]. Nevertheless, their potential to modify the immune response’s trajectory in RAS appears limited. In various studies, the supplementation of these deficient microelements only marginally influenced the disease progression. Conversely, positive outcomes were reported in response to oral vitamin B12 supplementation in RAS patients, independent of their initial serum levels of this microelement [9].

Regarding DDE, a review by Pastore et al. [61] states that children with celiac disease may experience a delay in tooth eruption and dental age, probably due to the growth retardation that is a common sign of the disease. In addition, an editorial [62] reported DDE as one of the oral manifestations of CD and highlighted possible orthodontic issues in patients who adhere to GFD late. Among the studies analyzed in this systematic review, only four articles dealt with DDE in patients with CD but with a higher prevalence than the controls. However, as also pointed out by Krzywicka et al. [62], the work of Mina et al. [37] found no significant differences in the eruption time of permanent teeth in celiac patients; indeed, in deciduous dentition, controls almost doubled the chance of a delayed eruption. Moreover, in the article by Moreau et al. [39], no patients with CD had a delay in eruption or maturation, whereas, in the control group, it was present in 3.57% of cases.

An editorial by Pastore et al. [63] presents the case of an atrophic tongue as the only clinical sign of CD. The authors also report the work of Lähteenoja et al. [33] in which the tongue was the most frequently affected site in a series of celiac patients. However, from the analysis of articles on the topic, atrophic glossitis was not the most frequently encountered clinical manifestation linked to CD. Furthermore, four of seven studies reported [11,33,35,36] that although the frequency of non-specific atrophic glossitis and geographic tongue is slightly higher, no significant difference was found with the control groups. Xerostomia and altered salivary flow rate seemed far more common. However, this oral manifestation has not been widely reported in literature reviews and meta-analyses [64,65].

Among subjective symptoms, pain and a burning sensation of the tongue have been reported [62]. It should be highlighted that glossodynia was reported in pediatric cohorts in three out of four studies [24,35,43].

Other oral manifestations found in the present literature review include OLP. A similar, shared autoimmune pathogenetic mechanism may underlie the associations between CD and other autoimmune diseases, such as OLP.

CD is an autoimmune disorder resulting from an inappropriate T cell-mediated immune response against ingested gluten. A breakdown of tolerance can lead to an aberrant immune response against self-peptides. The presence of only a genetic predisposition (HLA-DQ2.5, HLA-DQ8, or HLA-DQ2.2) cannot cause CD, so environmental risk factors are likely to play a critical role in the onset of the disease. Several studies have suggested that the microbiome may be crucial at the beginning of several autoimmune diseases [66,67,68,69,70]. Molecular mimicry, whereby T cells target microbial antigens that mimic particular (auto)antigens associated with autoimmune diseases, provides a model for the early phase of (auto)antigen sensitization by cross-reactive T cells [71,72]. The results of a 2020 study by Petersen et al. [67] show that molecular mimicry of an exogenous bacterial antigen may be a plausible primary mechanism contributing to the sudden onset of disease in HLA-DQ2+ individuals, usually in childhood, by inducing gluten-specific cross-reactive CD4+ T cells. Using a combined sequence homology search approach with structural and functional data on CD-associated T cells, the authors identified peptides from different bacteria that cross-reacted with T cells derived from CD patients. These data showed that common and abundant bacteria express highly active mimic antigens that closely resemble the immunodominant epitopes targeted by pathogenic T cells in CD.

In this context, the possibility that CD results from activating a pathogen-specific T cell response that cross-reacts with gluten epitopes seems plausible. Once this response is triggered, a gluten-driven expansion of the cross-reactive TCR repertoire and the spread of epitopes may follow; this may explain why only a minority of the gluten-specific T cell response induced by oral challenge with gluten responds to bacterial mimic epitopes. Finally, the high frequency of mimic peptides in our candidate peptide sample suggests that bacteria probably contain a pool of mimic antigens that fit into the context of other autoimmune diseases. This provides a rationale for the frequent association between CD and other autoimmune diseases [73].

## 5. Conclusions

CD has been linked to a broad spectrum of hard and soft tissue disorders within the oral cavity. Understanding this association could provide several benefits. Firstly, it could enhance the management of oral diseases through targeted CD therapy; secondly, it places healthcare professionals at the vanguard of patient management, particularly when dealing with cases where diagnosis is delayed.

## Figures and Tables

**Table 1:** First author and year of publication, PICOS information, and oral manifestation reported in the 27 articles described patients with RAS or a history of RAS.

Authors	Study Design	No. of Patients/ Controls	Age Patients/ Controls	Sex Patients/ Controls	Diagnosis	Outcome RAS No. (%)
Acar et al. (2012) [20]	Cross-sectional	35/ 35	13.7 (6–19)	17M;18F	CD	13 (37.1%)/4 (11.4%)
Ahmed et al. (2021) [21]	Cross-sectional	118/ 40	26 (20–37.23)/ 28.5 (21.3–36.8)	42M;76F 19M;21F	CD	44 (37.3%)/(12.5%)
Alsadat et al. (2021) [22]	Case-control	104/ 104	10.67 ± 2.39 (6–14) 10.69 ± 2.36	52M;52F 52M;52F	CD	44 (42.3%)/(15.4%)
Bijelić et al. (2019) [23]	Case-control	1			CD	1 RAS
Bramanti et al. (2014) [24]	Cross-sectional	50/ 54	7.5 ± 4.4/ 8.8 ±2.9	22M;28F 22M;32F	CD	26 (52%)/4 (7.4%)
Bucci et al. (2006) [25]	Case-control	72/ 162	9.05 ± 1.97	15M;57F 65M;97F	CD	24 (33.3%)/38 (23.4%)
Campisi et al. (2007) [26]	Case-control	197/ 413	19.09 (2–75)/ (2–77)	73M;124F 163M;250F	CD	37 (19%)/3 (1%)
Cruz et al. (2018) [27]	Case-control	40/ 40	16.50	12M;28F 28.7%M;71.3% F	CD	15 (43.9%)/32 (56.1%)
da Silva et al. (2008) [28]	Case report	1	39	1F	CD	1 (100%)
de Carvalho et al. (2015) [29]	Case-control	52/ 52	11.59± 5.74/ 11.48 (5.26)	18M;34F 23M;29F	CD	21 (40.38%)/9 (17.31)
Elbek-Cubukcu et al. (2023) [30]	Cross-sectional	62/ 64	9.17± 4.39 12.12± 3.3	20M;42F 20M;44F	CD	19 (30.6%)/0
Erriu et al. (2011) [31]	Observational study	98	35.92 (7–77)	23M;75F	CD	38 (38.77%)
Kovačić et al. (2021) [32]	Case report	1	41	1F	CD	1 (100%)
Lähteenoja et al. (1998) [33]	Cross-sectional	136/ 30	46.9 ± 9.8/ 47.9 ± 14.3	15M;15F 11M;19F	CD	1/0 RAS; 12/0 mucosal ulceration
Liu et al. (2022) [34]	Cross-sectional	20/ 20	49.2 ± 15.5 48.0 ± 12.7	4M;16F 2M;18F	CD	17 (85%)/7 (35%)
Ludovichetti et al. (2022) [35]	Retrospective cohort	38/ 38	(6 to 14)	11M;27F 12M;26F	CD	9 (23.7%)/3 (7.9%)
Macho et al. (2019) [36]	Case-control	80/ 80	13.3 (6 to 18)	32M;48F 35M;45F	CD	45 (56.3%)/16 (20%)
Mina et al. (2008) [37]	Case-control	52/ 23	7.9 (4–12)		CD	19 (63.15%)/0 (0%)
Mina et al. (2012) [38]	Observational longitudinal	25/ 25			CD	19 (63.15%)
Moreau et al. (2020) [39]	Retrospective cohort	28/ 59	8 (3 to 12)/ 7.2 (1 to 12)	9M;19F/ 30M;29F	CD	14 (50%)/12 (21.82%)
Nota et al. (2020) [40]	Structured questionnaire (survey)	237	(15 to 56)	55M;182F	CD	41 (17.3%)
Procaccini et al. (2007) [41]	Case-control	50/ 50	(3 to 25)		CD	18 (36%)/6 (12%)
Saraceno et al. (2016) [42]	Cross-sectional	83/83	9.15 (±2.12)	21M;62F	CD	58 (69%)
Shahraki et al. (2019) [43]	Case-control	65/ 60	8.9 (3–16)	23M;42F 31M;29F	CD	11 (17%)/8 (13%)
Yazdanbod et al. (2014) [44]	Cross-sectional	2	29	2F	CD	2 (100%) RAS
Yilmaz et al. (2020) [45]	Retrospective cohort	3			CD	3 (100%) RAS
Zoumpoulakis et al. (2019) [11]	Case-control	45/ 45	10.3 ± 4.1 10.3 ± 4.05	15M;30F	CD	18 (40%)/2 (4.4%)

**Table 2:** First author and year of publication, PICOS information, and oral manifestation reported in the 8 articles that report atrophic glossitis or geographic tongue among the manifestations of CD.

Authors	Study Design	No. of Patients/ Controls	Age Patients/ Controls	Sex Patients/ Controls	Diagnosis	Outcome No. (%)
Bramanti et al. (2014) [24]	Cross-sectional	50/ 54	7.5 ± 4.4/ 8.8 ± 2.9	22M;28F 22M;32F	CD	5 (10%)/2 (3.7%) geographic tongue; 7 (14%)/1 (1.85%) atrophic glossitis
Campisi et al. (2007) [26]	Case-control	197/ 413	19.09 (2–75)/ (2–77)	73M;124F 163M;250F	CD	31 (16%)/1 (0.2%) atrophic glossitis; 14 (7%)/5 (1%) geographic tongue
Lähteenoja et al. (1998) [33]	Cross-sectional	136/ 30	46.9 ± 9.8/ 47.9 ± 14.3	15M;15F 11M;19F	CD	4/1 atrophic tongue
Ludovichetti et al. (2022) [35]	Retrospective cohort	38/ 38	(6 to 14)	11M;27F 12M;26F	CD	8 (21%)/1 (26%) atrophic glossitis; 7 (18.4%)/3 (7.9%) geographic tongue
Macho et al. (2019) [36]	Case-control	80/ 80	13.3 (6 to 18)	32M;48F 35M;45F	CD	6 (7.5%)/1 (1.3%) geographic tongue; 5 (6.3%)/0 (0%) atrophic glossitis
Nota et al. (2020) [40]	Structured questionnaire (survey)	237	(15 to 56)	55M;182F	CD	12 (5.06%) glossitis
Procaccini et al. (2007) [41]	Case-control	50/ 50	(3 to 25)		CD	4 (8%)/1 (2%) atrophic glossitis
Zoumpoulakis et al. (2019) [11]	Case-control	45/ 45	10.3 ± 4.1 10.3 ± 4.05	15M;30F	CD	0 (0%)/0 (0%) atrophic glossitis; 3 (6.7%)/0 (0%) geographic tongue

**Table 3:** First author and year of publication, PICOS information, and oral manifestation reported in the 18 articles that reported dental enamel defects (DED).

Authors	Study Design	No. of Patients/ Controls	Age Patients/ Controls	Sex Patients/ Controls	Diagnosis	Outcome DED No. (%)
Acar et al. (2012) [20]	Cross-sectional	35	13.7 (6–19)	17M;18F	CD	14 (40%)/0
Ahmed et al. (2021) [21]	Cross-sectional	118/ 40	26 (20–37.23)/ 28.5 (21.3–36.8)	42M;76F 19M;21F	CD	79 (66.9%)/8 (20%)
Alsadat et al. (2021) [22]	Case-control	104/ 104	10.67 ± 2.39 (6–14) 10.69 ± 2.36	52M;52F 52M;52F	CD	73 (70.2%)/(34.6%)
Bramanti et al. (2014) [24]	Cross-sectional	50/ 54	7.5 ± 4.4/ 8.8 ± 2.9	22M;28F 22M;32F	CD	30 (60%)/0
Bucci et al. (2006) [25]	Case-control	72/ 162	9.05 ± 1.97	15M;57F 65M;97F	CD	14 (20%)/9 (5.55%)
Campisi et al. (2007) [26]	Case-control	197/ 413	19.09 (2–75)/ (2–77)	73M;124F 163M;250F	CD	46 (23%)/(9%)
Cruz et al. (2018) [27]	Case-control	40/ 40	16.50	12M;28F 28.7%M;71.3% F	CD	15 (65.2%)/8 (34.8%)
de Carvalho et al. (2015) [29]	Case-control	52/ 52	11.59 ± 5.74/ 11.48 (5.26)	18M;34F 23M;29F	CD	32 (61.54%)/11 (21.15%)
Elbek-Cubukcu et al. (2023) [30]	Cross-sectional	62/ 64	9.17 ± 4.39 12.12 ± 3.3	20M;42F 20M;44F	CD	38 (61%)/42 (65.6%) MIH
Erriu et al. (2011) [31]	Observational study	98	35.92 (7–77)	23M;75F	CD	28 (28.57%)
Ludovichetti et al. (2022) [35]	Retrospective cohort	38/ 38	(6 to 14)	11M;27F 12M;26F	CD	26/11
Macho et al. (2020) [46]	Case-control	80/ 80	6 to 18	32M;48F 35M;45F	CD	44 (55%)/22 (27.5)
Mina et al. (2012) [38]	Observational longitudinal	25/ 25			CD	(30%)
Moreau et al. (2020) [39]	Retrospective cohort	28/ 59	8 (3 to 12)/ 7.2 (1 to 12)	9M;19F/ 30M;29F	CD	19 (67.86%)/20 (33.9%)
Procaccini et al. (2007) [41]	Case-control	50	(3 to 25)		CD	13 (26%)/8 (16%)
Saraceno et al. (2016) [42]	Cross-sectional	83/83	9.15 (±2.12)	21M;62F	CD	10 DED (12%)
Shahraki et al. (2019) [43]	Case-control	65/ 60	8.9 (3–16)	23M;42F 31M;29F	CD	38 (58.46%)/14 (23.3%)
Zoumpoulakis et al. (2019) [11]	Case-control	45/ 45	10.3 ± 4.1 10.3 ± 4.05	15M;30F	CD	29 (64.4%)/11 (24.4%)

**Table 4:** First author and year of publication, PICOS information, and oral manifestation reported in the 4 articles that reported delayed dental eruption (DEE).

Authors	Study Design	No. of Patients/ Controls	Age Patients/ Controls	Sex Patients/ Controls	Diagnosis	Outcome DDE No. (%)
Alamoudi et al. (2020) [47]	Case-control	104/104	10.67 ± 2.40/ 10.69 ± 2.37	52M;52F 52M;52F	CD	65 (62.5%)/3 (2.9%)
Bramanti et al. (2014) [24]	Cross-sectional	50/ 54	7.5 ± 4.4/ 8.8 ± 2.9	22M;28F 22M;32F	CD	24 (48%)/0
Mina et al. (2008) [37]	Case-control	52/ 23	7.9 (4–12)		CD	(32%)/(38%)
Moreau et al. (2020) [39]	Retrospective cohort	28/ 59	8 (3 to 12)/ 7.2 (1 to 12)	9M;19F/ 30M;29F	CD	0 (0%)/2 (3.57%)

**Table 5:** First author and year of publication, PICOS information, and oral manifestation reported in the 7 articles that reported xerostomia among the intraoral manifestations of CD.

Authors	Study Design	No. of Patients/ Controls	Age Patients/ Controls	Sex Patients/ Controls	Diagnosis	Outcome Xerostomia No. (%)
Ahmed et al. (2021) [21]	Cross-sectional	118/ 40	26 (20–37.23)/ 28.5 (21.3–36.8)	42M;76F 19M;21F	CD	81 (68.6%)/3 (7.5%)
Cruz et al. (2018) [27]	Case-control	40/ 40	16.50	12M;28F 28.7M;71F	CD	13 (86.7%)/2 (13.3%)
de Carvalho et al. (2015) [29]	Case-control	52/ 52	11.59 ± 5.74/ 11.48 (5.26)	18M;34F 23M;29F	CD	18 (36%)/6 (12%)
Lähteenoja et al. (1998) [33]	Cross-sectional	136/ 30	46.9 ± 9.8/ 47.9 ± 14.3	15M;15F 11M;19F	CD	3 (10%)/7 (23%)
Liu et al. (2022) [34]	Cross-sectional	20/ 20	49.2 ± 15.5 48.0 ± 12.7	4M;16F 2M;18F	CD	12 (65%)/0 (0%)
Nota et al. (2020) [40]	Structured questionnaire (survey)	237	(15 to 56)	55M;182F	CD	38 (16.45%)
Shahraki et al. (2019) [43]	Case-control	65/ 60	8.9 (3–16)	23M;42F 31M;29F	CD	10 (15.4%)/3 (5%)

**Table 6:** First author and year of publication, PICOS information, and oral manifestation reported in the 4 studies that described glossodynia.

Authors	Study Design	No. of Patients/ Controls	Age Patients/ Controls	Sex Patients/ Controls	Diagnosis	Outcome Glossodynia No. (%)
Bramanti et al. (2014) [24]	Cross-sectional	50/ 54	7.5 ± 4.4/ 8.8 ± 2.9	22M;28F 22M;32F	CD	7 (14%)/3 (5.55%)
Lucchese et al. (2012) [48]	Case report	1	72	1F	CD	1 (100%)
Ludovichetti et al. (2022) [35]	Retrospective cohort	38/ 38	(6 to 14)	11M;27F 12M;26F	CD	6 (15.8%)/0 (0%)
Shahraki et al. (2019) [43]	Case-control	65/ 60	8.9 (3–16)	23M;42F 31M;29F	CD	8 (12.31%)/2 (3%)

**Table 7:** First author and year of publication, PICOS information, and oral manifestation reported in the 10 articles that described other oral manifestations.

Authors	Study Design	No. of Patients/ Controls	Age Patients/ Controls	Sex Patients/ Controls	Diagnosis	Outcome No. (%)
Ahmed et al. (2021) [21]	Cross-sectional	118/ 40	26 (20–37.23)/ 28.5 (21.3–36.8)	42M;76F 19M;21F	CD	16 (13.6%)/0 (0%) cheilitis
Bramanti et al. (2014) [24]	Cross-sectional	50/ 54	7.5 ± 4.4/ 8.8 ± 2.9	22M;28F 22M;32F	CD	3 (6%)/2 (3.7%) angular cheilitis
Družijanic et al. (2019) [49]	Cross-sectional	7			CD	(11.11%) oral lichen planus
Kustro et al. (2020) [50]	Observational study	25	41.03 (18 to 50)		CD	40 (80%) dystrophic inflammatory periodontal diseases
Liu et al. (2022) [34]	Cross-sectional	20/ 20	49.2 ± 15.5 48.0 ± 12.7	4M;16F 2M;18F	CD	7 (35%)/0 (0%) dry and cracking lips; 4 (20%)/0 (0%) mucosal erythema; 1 (5%)/0 (0%) denture stomatitis; 4 (20%)/2 (10%) fissured tongue; 7 (35%)/2 (10%) oral mucosal itching and burning sensation; 8 (40%)/3 (15%) taste disturbances
Lucchese et al. (2012) [48]	Case report	1	72	1F	CD	1 (100%) fissured tongue
Ludovichetti et al. (2022) [35]	Retrospective cohort	38/ 38	(6 to 14)	11M;27F 12M;26F	CD	4 (10.5%)/0 (0%) angular cheilitis
Macho et al. (2019) [36]	Case-control	80/ 80	13.3 (6 to 18)	32M;48F 35M;45F	CD	5 (6.3%)/0 (0%) angular cheilitis.
Nota et al. (2020) [40]	Structured questionnaire (survey)	237	(15 to 56)	55M;182F	CD	18 (7.59%) gingivitis; 12 (5.06%) glossitis
Saalman et al. (2008) [51]	Prospective cohort	1	11	1M	CD	Orofacial granulomatosis

## Data Availability

Not applicable.

## Supplementary Materials

The following supporting information can be downloaded at: https://www.mdpi.com/article/10.3390/jcm12123874/s1.

## References

[1] Caio G., Volta U., Sapone A., Leffler D.A., de Giorgio R., Catassi C., Fasano A. (2019). Celiac Disease: A Comprehensive Current Review. BMC Med..

[2] Kagnoff M.F. (2007). Celiac Disease: Pathogenesis of a Model Immunogenetic Disease. J. Clin. Investig..

[3] Catassi C., Bai J.C., Bonaz B., Bouma G., Calabrò A., Carroccio A., Castillejo G., Ciacci C., Cristofori F., Dolinsek J. (2013). Non-Celiac Gluten Sensitivity: The New Frontier of Gluten Related Disorders. Nutrients.

[4] Picarelli A., di Tola M., Borghini R., Isonne C., Saponara A., Marino M., Casale R., Tiberti A., Pica R., Donato G. (2013). Colonic Involvement in Celiac Disease and Possible Implications of the Sigmoid Mucosa Organ Culture in Its Diagnosis. J. Clin. Immunol..

[5] Liu E., Lee H.-S., Aronsson C.A., Hagopian W.A., Koletzko S., Rewers M.J., Eisenbarth G.S., Bingley P.J., Bonifacio E., Simell V. (2014). Risk of Pediatric Celiac Disease According to HLA Haplotype and Country. N. Engl. J. Med..

[6] McAllister B.P., Williams E., Clarke K. (2019). A Comprehensive Review of Celiac Disease/Gluten-Sensitive Enteropathies. Clin. Rev. Allergy Immunol..

[7] Werkstetter K.J., Korponay-Szabó I.R., Popp A., Villanacci V., Salemme M., Heilig G., Lillevang S.T., Mearin M.L., Ribes-Koninckx C., Thomas A. (2017). Accuracy in Diagnosis of Celiac Disease Without Biopsies in Clinical Practice. Gastroenterology.

[8] Garnier-Lengliné H., Cerf-Bensussan N., Ruemmele F.M. (2015). Celiac Disease in Children. Clin. Res. Hepatol. Gastroenterol..

[9] Baccaglini L., Lalla R., Bruce A., Sartori-Valinotti J., Latortue M., Carrozzo M., Rogers R. (2011). Urban Legends: Recurrent Aphthous Stomatitis. Oral Dis..

[10] Laurikka P., Nurminen S., Kivelä L., Kurppa K. (2018). Extraintestinal Manifestations of Celiac Disease: Early Detection for Better Long-Term Outcomes. Nutrients.

[11] Zoumpoulakis M., Fotoulaki M., Topitsoglou V., Lazidou P., Zouloumis L., Kotsanos N. (2019). Prevalence of Dental Enamel Defects, Aphthous-like Ulcers and Other Oral Manifestations in Celiac Children and Adolescents: A Comparative Study. J. Clin. Pediatr. Dent..

[12] Costacurta M., Maturo P., Bartolino M., Docimo R. (2010). Oral Manifestations of Coeliac Disease.: A Clinical-Statistic Study. Oral Implant..

[13] Nieri M., Tofani E., Defraia E., Giuntini V., Franchi L. (2017). Enamel Defects and Aphthous Stomatitis in Celiac and Healthy Subjects: Systematic Review and Meta-Analysis of Controlled Studies. J. Dent..

[14] Hasan A., Patel H., Saleh H., Youngberg G., Litchfield J., Krishnaswamy G. (2013). Remission of Severe Aphthous Stomatitis of Celiac Disease with Etanercept. Clin. Mol. Allergy.

[15] Rivera E., Assiri A., Guandalini S. (2013). Celiac Disease. Oral Dis..

[16] Grassia V., Gentile E., di Stasio D., Jamilian A., Matarese G., D’Apuzzo F., Santoro R., Perillo L., Serpico R., Lucchese A. (2016). In Vivo Confocal Microscopy Analysis of Enamel Defects after Orthodontic Treatment: A Preliminary Study. Ultrastruct. Pathol..

[17] Compilato D., Campisi G., Pastore L., Carroccio A. (2010). The Production of the Oral Mucosa of Antiendomysial and Anti-Tissue-Transglutaminase Antibodies in Patients with Celiac Disease: A Review. Sci. World J..

[18] Pastore L., Campisi G., Compilato D., lo Muzio L. (2008). Orally Based Diagnosis of Celiac Disease: Current Perspectives. J. Dent. Res..

[19] Hinneburg I. (2017). ROBINS-1: A Tool for Asssessing Risk of Bias in Non-Randomised Studies of Interventions. Med. Mon. Pharm..

[20] Acar S., Yetkiner A.A., Ersin N., Oncag O., Aydogdu S., Arikan C. (2012). Oral Findings and Salivary Parameters in Children with Celiac Disease: A Preliminary Study. Med. Princ. Pract..

[21] Ahmed A., Singh A., Kajal S., Chauhan A., Rajput M.S., Banyal V., Ahuja V., Makharia G.K. (2021). Dental Enamel Defects and Oral Cavity Manifestations in Asian Patients with Celiac Disease. Indian J. Gastroenterol..

[22] Alsadat F.A., Alamoudi N.M., El-Housseiny A.A., Felemban O.M., Dardeer F.M., Saadah O.I. (2021). Oral and Dental Manifestations of Celiac Disease in Children: A Case–Control Study. BMC Oral Health.

[23] Bijelić B., Matić I.Z., Besu I., Janković L., Juranić Z., Marušić S., Andrejević S. (2019). Celiac Disease-Specific and Inflammatory Bowel Disease-Related Antibodies in Patients with Recurrent Aphthous Stomatitis. Immunobiology.

[24] Bramanti E., Cicciù M., Matacena G., Costa S., Magazzù G. (2014). Clinical Evaluation of Specific Oral Manifestations in Pediatric Patients with Ascertained versus Potential Coeliac Disease: A Cross-Sectional Study. Gastroenterol. Res. Pr..

[25] Bucci P., Carile F., Sangianantoni A., D’Angiò F., Santarelli A., Muzio L. (2006). Lo Oral Aphthous Ulcers and Dental Enamel Defects in Children with Coeliac Disease. Acta Paediatr. Int. J. Paediatr..

[26] Campisi G., Di Liberto C., Iacono G., Compilato D., Di Prima L., Calvino F., Di Marco V., Lo Muzio L., Sferrazza C., Scalici C. (2007). Oral Pathology in Untreated Coelic Disease. Aliment. Pharm..

[27] Izabela-Taiatella-Siqueira-Alves C., Fraiz F.C., Celli A., Amenabar J.M., Luciana-Reichert-Da-Silva A. (2018). Dental and Oral Manifestations of Celiac Disease. Med. Oral Patol. Oral Cir. Bucal..

[28] da Silva P.C., de Almeida P.D.V., Machado M.A.N., de Lima A.A.S., Grégio A.M.T., Trevilatto P.C., Azevedo-Alanis L.R. (2008). Oral Manifestations of Celiac Disease. A Case Report and Review of the Literature. Med. Oral Patol. Oral Cir. Bucal..

[29] De Carvalho F.K., De Queiroz A.M., Bezerra Da Silva R.A., Sawamura R., Bachmann L., Bezerra Da Silva L.A., Nelson-Filho P. (2015). Oral Aspects in Celiac Diseas.s.se Children: Clinical and Dental Enamel Chemical Evaluation. Oral Surg. Oral Med. Oral Pathol. Oral Radiol..

[30] Elbek-Cubukcu C., Arsoy H.A., Ozkaya G. (2023). Assessment of Oral Manifestations in Pediatric Patients with Celiac Disease in Relation to Marsh Types. Med. Oral Patol. Oral Cir. Bucal..

[31] Erriu M., Sanna S., Nucaro A., Orrù G., Garau V., Montaldo C. (2011). HLA-DQB1 Haplotypes and Their Relation to Oral Signs Linked to Celiac Disease Diagnosis. Open Dent. J..

[32] Kovacic M., Kovacic I., Versic M. (2021). Necrotic Ulcerative Stomatitis in a Patient with Long-Standing Celiac Disease: A Case Report. Croat. Med. J..

[33] Lähteenoja H., Toivanen A., Viander M., Mäki M., Irjala K., Räihä I., Syrjänen S. (1998). Oral Mucosal Changes in Coeliac Patients on a Gluten-Free Diet. Eur. J. Oral Sci..

[34] Liu J., Lundemann A.K.J., Reibel J., Pedersen A.M.L. (2022). Salivary Gland Involvement and Oral Health in Patients with Coeliac Disease. Eur. J. Oral Sci..

[35] Ludovichetti F.S., Signoriello A.G., Girotto L., Del Dot L., Piovan S., Mazzoleni S. (2022). Oro-Dental Lesions in Paediatric Patients with Coeliac Disease: An Observatioanl Retrospective Clinical Study. Rev. Española De Enferm. Dig..

[36] MacHo V., Manso M., Silva D., Andrade D. (2019). Does the Introduction of Gluten-Free Diet Influence the Prevalence of Oral Soft Tissue Lesions in Celiac Disease?. J. Int. Oral Health.

[37] Mina S.S., Azcurra A.I., Dorronsoro S., Brunotto M.N. (2008). Alterations of the oral ecosystem in children with celiac disease. Acta Odontol. Latinoam..

[38] Mina S., Riga C., Azcurra A.I., Brunotto M. (2012). Oral Ecosystem Alterations in Celiac Children: A Follow-up Study. Arch. Oral Biol..

[39] Villemur Moreau L., Dicky O., Mas E., Noirrit E., Marty M., Vaysse F., Olives J.P. (2021). Oral Manifestations of Celiac Disease in French Children. Arch. De Pediatr..

[40] Nota A., Abati S., Bosco F., Rota I., Polizzi E., Tecco S. (2020). General Health, Systemic Diseases and Oral Status in Adult Patients with Coeliac Disease. Nutrients.

[41] Procaccini M., Campisi G., Bufo P., Compilato D., Massaccesi C., Catassi C., Lo Muzio L. (2007). Lack of Association between Celiac Disease and Dental Enamel Hypoplasia in a Case-Control Study from an Italian Central Region. Head Face Med..

[42] Saraceno R., Perugia C., Ventura A., LORè B., Chimenti S., Docimo R. (2016). Aphthous, Celiac Disease and Other Dental Disorders in Childhood. G Ital. Derm. Venereol..

[43] Shahraki T., Mehr S.O., Hill I.D., Shahraki M. (2019). A Comparison of the Prevalence of Dental Enamel Defects and Other Oral Findings in Children with and without Celiac Disease. Iran. J. Pediatr..

[44] Yazdanbod A., Nemati R., Alamdari M.I., Azami A., Maleki N. (2014). Prevalence of Celiac Disease in Patients with Recurrent Aphthous Stomatitis. Govaresh.

[45] Yılmaz S., Tuna Kırsaçlıoğlu C., Şaylı T.R. (2020). Celiac Disease and Hematological Abnormalities in Children with Recurrent Aphthous Stomatitis. Pediatr. Int..

[46] Macho V.M.P., de Barros Menéres Manso M.C.A., e Silva D.M.V., de Andrade D.J.C. (2020). The Difference in Symmetry of the Enamel Defects in Celiac Disease versus Non-Celiac Pediatric Population. J. Dent. Sci..

[47] Alamoudi N.M., Alsadat F.A., El-Housseiny A.A., Felemban O.M., al Tuwirqi A.A., Mosli R.H., Saadah O.I. (2020). Dental Maturity in Children with Celiac Disease: A Case–Control Study. BMC Oral Health.

[48] Lucchese A., Guida A., Serpico R. (2012). Glossodynia and Coeliac Disease. Immunopharmacol. Immunotoxicol..

[49] Družijanic A., Glavina A., Draganja M., Biočina-Lukenda D., Cigic L. (2019). Inflammatory Markers and Incidence of Other Autoimmune Diseases in Patients with Oral Lichen Planus. Acta Stomatol. Croat..

[50] Kustro T., Antonenko M., Gubska O. (2020). Clinicoradiologic Aspects of Periodontal Diseases in Patients with Gluten-Related Disorders. Balneo Res. J..

[51] Saalman R., Mattsson U., Jontell M. (2009). Orofacial Granulomatosis in Childhood—A Clinical Entity That May Indicate Crohn’s Disease as Well as Food Allergy. Acta Paediatr. Int. J. Paediatr..

[52] Aine L., Mäki M., Collin P., Keyriläinen O. (1990). Dental Enamel Defects in Celiac Disease. J. Oral Pathol. Med..

[53] Souto-Souza D., da Consolação Soares M.E., Rezende V.S., de Lacerda Dantas P.C., Galvão E.L., Falci S.G.M. (2018). Association between Developmental Defects of Enamel and Celiac Disease: A Meta-Analysis. Arch. Oral Biol..

[54] Wieser H., Amato M., Caggiano M., Ciacci C. (2023). Dental Manifestations and Celiac Disease&mdash;An Overview. J. Clin. Med..

[55] Petronijevic S., Stig S., Gao J., Halstensen T.S. (2016). Amelogenin Specific IgA and IgG in Children with Untreated Coeliac Disease. Eur. J. Oral Sci..

[56] Freeman H.J. (2010). Risk Factors in Familial Forms of Celiac Disease. World J. Gastroenterol..

[57] Ålebioda Z., Szponar E., Kowalska A. (2014). Etiopathogenesis of Recurrent Aphthous Stomatitis and the Role of Immunologic Aspects: Literature Review. Arch. Immunol. Exp..

[58] Xiao X., Deng Y., Long Y., Liu W., Shi H. (2023). Evaluation of Cytokines as Diagnostic and Therapeutic Indicators for Recurrent Aphthous Stomatitis: A Statistical Study. J. Dent. Sci..

[59] Sanchez-Solares J., Sanchez L., Pablo-Torres C., Diaz-Fernandez C., Sørensen P., Barber D., Gomez-Casado C. (2021). Celiac Disease Causes Epithelial Disruption and Regulatory T Cell Recruitment in the Oral Mucosa. Front. Immunol..

[60] Bao Z.X., Shi J., Yang X.W., Liu L.X. (2018). Hematinic Deficiencies in Patients with Recurrent Aphthous Stomatitis: Variations by Gender and Age. Med. Oral Patol. Oral Cir. Bucal..

[61] Pastore L., Carroccio A., Compilato D., Panzarella V., Serpico R., Lo Muzio L. (2008). Oral Manifestations of Celiac Disease. J. Clin. Gastroenterol..

[62] Krzywicka B., Herman K., Kowalczyk-Zając M., Pytrus T. (2014). Celiac Disease and Its Impact on the Oral Health Status—Review of the Literature. Adv. Clin. Exp. Med..

[63] Pastore L., Lo Muzio L., Serpico R. (2007). Atrophic Glossitis Leading to the Diagnosis of Celiac Disease. N. Engl. J. Med..

[64] Van Gils T., Brand H.S., De Boer N.K.H., Mulder C.J.J., Bouma G. (2017). Gastrointestinal Diseases and Their Oro-Dental Manifestations: Part 3: Coeliac Disease. Br. Dent. J..

[65] della Vella F., Lauritano D., Lajolo C., Lucchese A., di Stasio D., Contaldo M., Serpico R., Petruzzi M. (2019). The Pseudolesions of the Oral Mucosa: Differential Diagnosis and Related Systemic Conditions. Appl. Sci..

[66] Ciacchi L., Reid H.H., Rossjohn J. (2022). Structural Bases of T Cell Antigen Receptor Recognition in Celiac Disease. Curr. Opin. Struct. Biol..

[67] Petersen J., Ciacchi L., Tran M.T., Loh K.L., Kooy-Winkelaar Y., Croft N.P., Hardy M.Y., Chen Z., McCluskey J., Anderson R.P. (2020). T Cell Receptor Cross-Reactivity between Gliadin and Bacterial Peptides in Celiac Disease. Nat. Struct. Mol. Biol..

[68] Lucchese A. (2019). Periodontal Bacteria and the Rheumatoid Arthritis-Related Antigen RA-A47: The Cross-Reactivity Potential. Curr. Opin. Rheumatol..

[69] Nikitakis N.G., Papaioannou W., Sakkas L.I., Kousvelari E. (2017). The Autoimmunity-Oral Microbiome Connection. Oral Dis..

[70] Shaheen W.A., Quraishi M.N., Iqbal T.H. (2022). Gut Microbiome and Autoimmune Disorders. Clin. Exp. Immunol..

[71] Mittelman A., Lucchese A., Sinha A.A., Kanduc D. (2002). Monoclonal and Polyclonal Humoral Immune Response to EC HER-2/NEU Peptides with Low Similarity to the Host’s Proteome. Int. J. Cancer.

[72] Kanduc D., Lucchese A., Mittelman A. (2007). Non-Redundant Peptidomes from DAPs: Towards “the Vaccine”?. Autoimmun. Rev..

[73] Neuhausen S.L., Steele L., Ryan S., Mousavi M., Pinto M., Osann K.E., Flodman P., Zone J.J. (2008). Co-Occurrence of Celiac Disease and Other Autoimmune Diseases in Celiacs and Their First-Degree Relatives. J. Autoimmun..

