# Supplementary figures and images for: Beyond the Gut: A Systematic Review of Oral Manifestations in Celiac Disease
Source: J Clin Med. 2023 Jun 6;12(12):3874. doi: 10.3390/jcm12123874 (PMC10299058; doi:10.3390/jcm12123874)

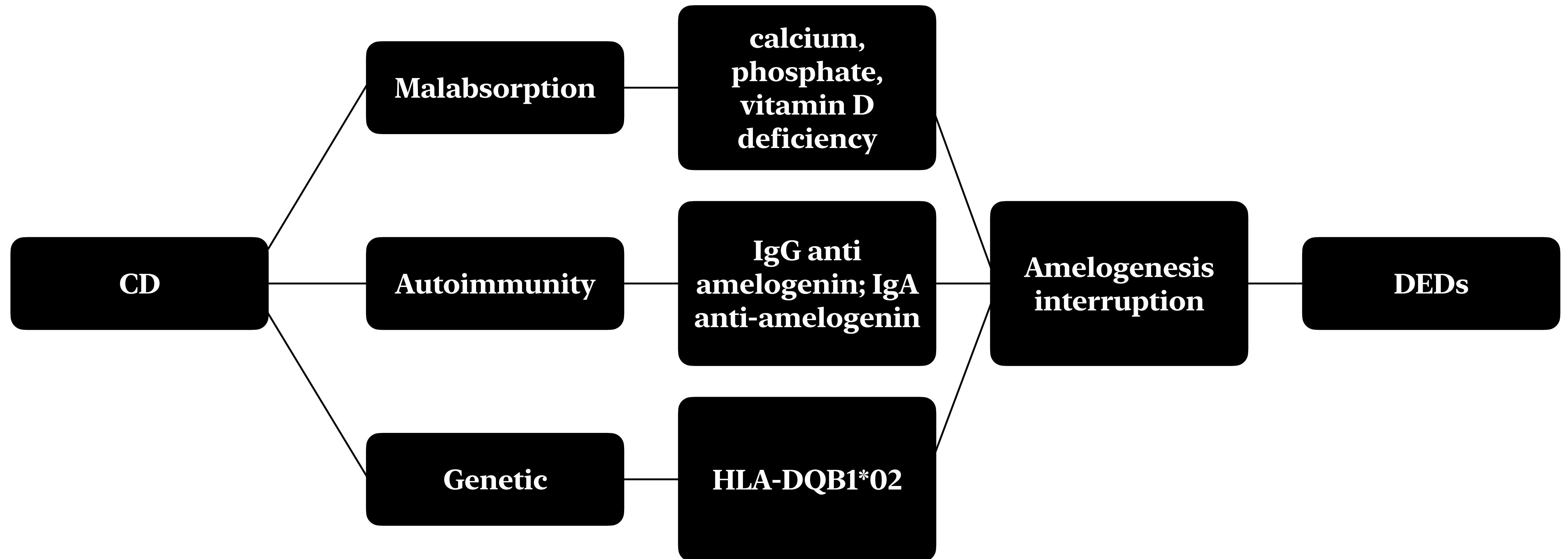

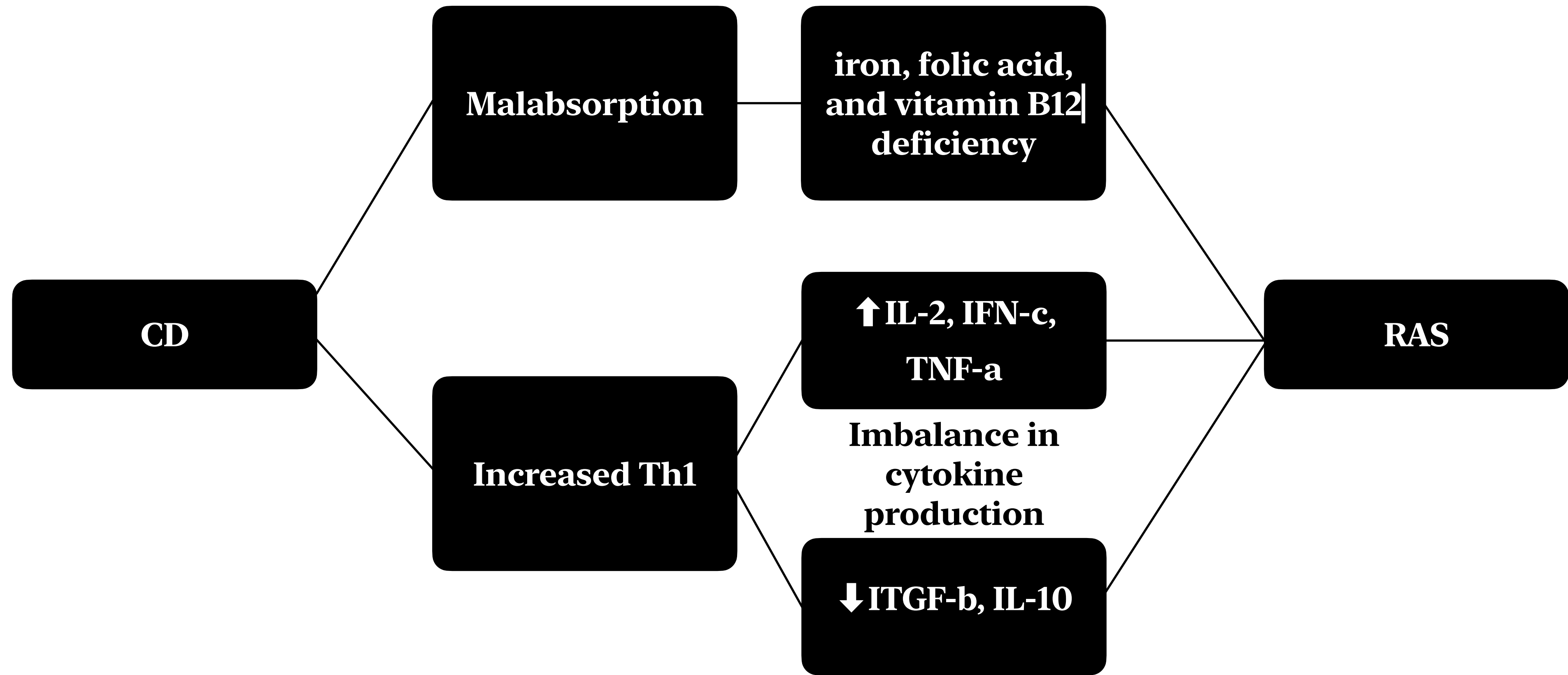

Supplement: Supplementary file 1 [file jcm-12-03874-s001.zip › jcm-2259051-supplementary.pdf]
